# Single-step extraction for simultaneous quantification of desvenlafaxine and alprazolam in human spiked plasma by RP-HPLC

**DOI:** 10.1371/journal.pone.0238954

**Published:** 2020-09-17

**Authors:** Huma Rao, Saeed Ahmad, Asadullah Madni, Imtiaz Ahmad, Muhammad Nadeem Shahzad

**Affiliations:** Faculty of Pharmacy, The Islamia University of Bahawalpur, Bahawalpur, Pakistan; Bhagwan Mahvir College of Pharmacy, INDIA

## Abstract

Desvenlafaxine (DES) and Alprazolam (ALP) are the drugs commonly prescribed together for the treatment of Major Depressive Disorders (MDD). A literature survey revealed, there is no method for the simultaneous determination of these two drugs. The purpose of this research was to develop and validate a simple, accurate, precise, robust, and isocratic RP–HPLC method for simultaneous determination of DES and ALP in human spiked plasma using UV-detector in short analysis time. The method utilized Hypersil BDS C18 (250 mm×4.6 mm, 5 μm) through an isocratic mode of elution using HPLC grade acetonitrile and 0.02M KH_2_PO_4_ buffer (65:35) and 0.1% Tri Fluoro Acetic acid (TFA) with pH 4.00 adjusted with 1M KOH. The flow rate was 1.00 mLmin^-1^ and elution of the drugs was monitored at 230nm. The elution time of DES and ALP was 4.011 and 5.182 minutes respectively. The method was linear for the concentration range 10–150 μgmL^-1^ for DES and 5.0–75.0 μgmL^-1^ for ALP. According to the validation results, the method is sensitive with Limit of Detection (LOD) 4.740 μgmL^-1^ and Limit of Quantification (LOQ) of 14.365 μgmL^-1^ for DES and LOD 1.891 μgmL^-1^ & LOQ 5.730 μgmL^-1^ for ALP. The reproducibility of results with minute deliberate variations in method parameters has proven that the method is robust. The data from stability studies show a non-significant change in drugs solutions for 2 months. The optimized method was validated as per International Conference for Harmonisation (ICH) Q2(R1) guidelines. This method can be used for the estimation of DES and ALP in plasma and can evaluate pharmacokinetic parameters of both drugs simultaneously.

## Introduction

Depression refers to a heterogeneous group of ailments varying not only in symptoms but also in etiology. It may express itself either as major depressive disorder or a minor disease with a collection of physical as well as mental depressive symptoms. In 2015, WHO estimated that 4.4% of the global population is affected with depression but now it is ranked 4^th^ among global causes of disease burden as approximately 17% of the world population experiences an episode of MDD during lifetime [1–3]. Its prevalence rate peaks in old adulthood with >7.5% in females and >5.5% in males. It also occurs in children and adolescents but at a lower rate as compared to adults [3].

The evidence base for the pharmacologic treatment of MDD is currently greatest for selective serotonin reuptake inhibitors (SSRIs), serotonin-norepinephrine reuptake inhibitors (SNRIs), and benzodiazepines. SNRIs and benzodiazepines are the two most widely prescribed classes of medications for major depressive disorders (MDD), as the co-occurrence of depression and anxiety with associated sleep disturbances are common in the clinical scenario. This Combination therapy with SNRIs and benzodiazepines may improve outcomes in appropriate clinical settings as compared to monotherapy prescribed to the patients [4–6].

SNRIs are USFDA approved drugs for the treatment of MDD. However, they have a slow onset of action and the patient may take several weeks for improvement in his clinical conditions. Also, they cause symptoms worsening initially. Benzodiazepines have a rapid onset of action and have high patient acceptance due to its efficiency in the management of acute exacerbation of anxiety symptoms [1, 2].

A combination of SNRIs and benzodiazepines can offer the patients rapid control of anxiety and sleep disturbances, lessened frequency of SNRI induced anxiety and agitation that occurs early in course of therapy, and hence enhanced adherence to the course of therapy [3].

**Fig 1:**
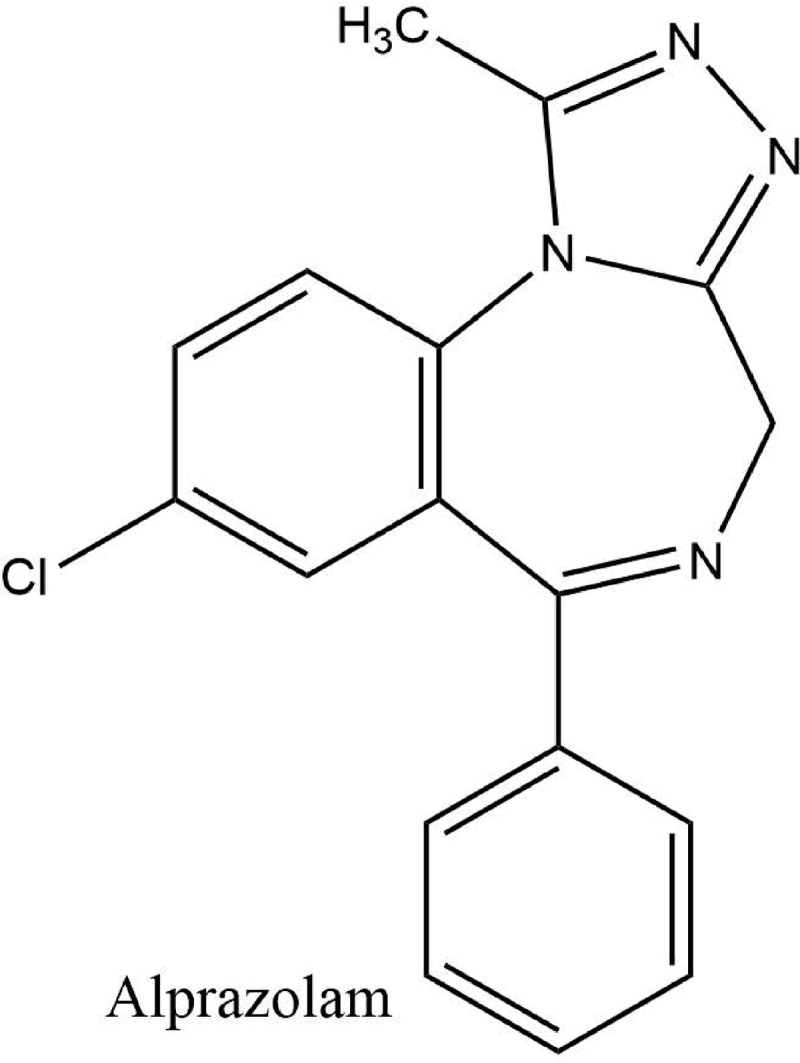
Structure of desvenlafaxine.

Desvenlafaxine (DES) is the third SNRI approved by USFDA in 2009 for the treatment of MDD. Chemically it is 4-[2-(dimethylamino)-1-(1-hydroxycyclohexyl) ethyl] phenol [4, 5] as shown in Fig 1. It is available as extended-release tablets of 50mg and 100mg and is one of the preferred SNRIs used in depression [7, 8].

**Fig 2:**
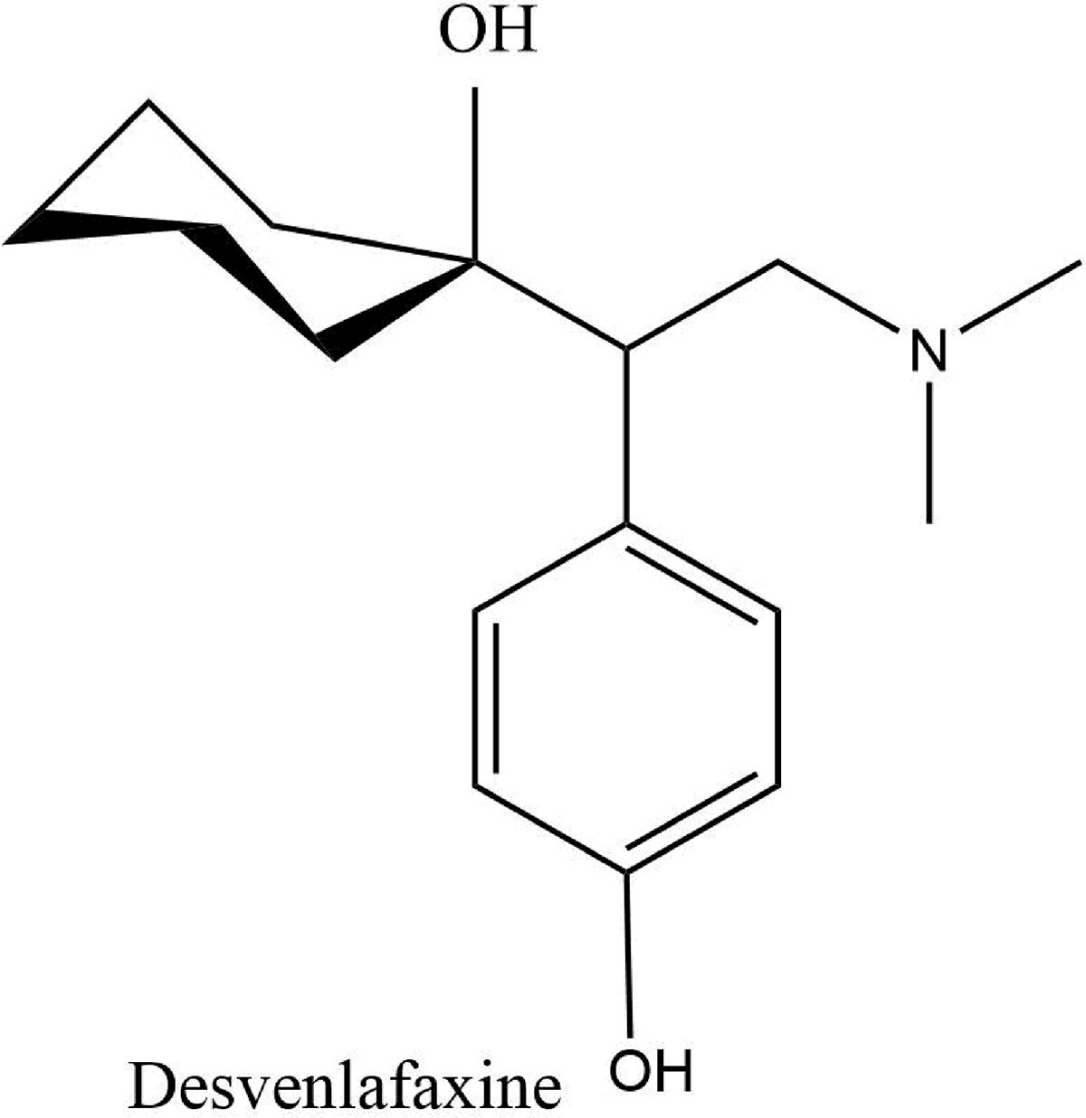
Structure of alprazolam.

Alprazolam (8-chloro-1-methyl-6-phenyl-4H-[1,2,4] triazolo [4,3,-α]-[1,4] benzodiazepine) is a fifth-generation benzodiazepine derived from 1,4-benzodiazepines [9]. In alprazolam, the addition of 1,2,4-triazole ring at 1,2 position of diazepine has imparted antidepressant activity to this drug [10, 11] as shown in Fig 2. Due to the fast onset of action and less dose, it offers a safe and potent clinical option to relieve anxiety and sleep disturbances associated with depression in dose 0.5–3.0 mg^-1^day [5, 12]. DES and ALP are used in combination for depression to provide rapid control of depressive symptoms due to the faster onset of action of ALP. Combined prescription of both drugs concomitantly offers a good therapeutic choice for the treatment of depression as it offers tolerability, convenience, cost-effectiveness, and compliance.

Most of the analytical procedures discussed in the literature for the determination of DES or ALP, either independently or along with other antidepressant drugs, involve Spectrophotometry [13–15], as well as Liquid chromatography techniques including HPLC-UV [16–25], LC-MS [7, 25], LC-MS/MS [26], LC-ESI-MS/MS [27]. Most of the conventional analytical laboratories lack expensive instrumentation used in LC-MS. Chromatographic methods for the determination of ALP have been reported in official compendia, whereas no official method is available for quantification of DES in the dosage forms and biological fluids. The reported methods utilize a gradient mobile phase and a very complex multi-step extraction procedure for plasma sample preparation that make the analysis costly and tedious.

Thus, it was necessary to develop a simple, economical, precise, accurate, and rapid method that is based on single-step protein precipitation for sample preparation and use HPLC with simple UV detection for simultaneous quantification of DES & ALP in human spiked plasma. This method quantifies both drugs simultaneously in human spiked plasma in a single chromatographic run with shorter run time. This study describes validation parameters stated by ICH Q2R1 guidelines [28] to ensure suitability, accuracy, reliability as well as the feasibility of the developed method.

## Materials and methods

### Chemicals & reagents

Reference drug substance of desvenlafaxine (DES) & alprazolam (ALP) were kindly provided by Genix Pharma (Private) Ltd Karachi, Pakistan as a donation for research work. All chemicals and reagents used in this research were of analytical grade, whereas all solvents used were of HPLC grade. Methanol, acetonitrile, and ortho-phosphoric acid were procured from Merck, Darmstadt, Germany. Monobasic potassium dihydrogen phosphate and potassium hydroxide were obtained from Sigma Aldrich Corporation, USA.

### Equipment

The mobile phase was degassed using Elmasonic sonicator (E 30 H) Elma Schmidbauer GmbH, Germany. A digital pH meter (InoLab WTW Series) was used for pH measurement and adjustment. A magnetic stirrer by Velp Scientifica, USA was used for the preparation of buffer solution and mobile phase. Filtration Assembly, Merck Millipore, Germany was utilized for filtration. The mobile phase was filtered by Durapore PVDF membrane filters with a diameter of 47mm & 0.45μm pore size, procured from Merck, Massachusetts, USA. Sample solutions were filtered through nylon syringe filters with 13mm & 25mm diameter and 0.45μm pore size, procured from CNW Technologies, Duesseldorf, Germany.

### Instrument

The separation was achieved on HPLC Perkin Elmer series 200, USA equipped with a Series 200 LC- pump, a manual Rheodyne injector valve of 20 μL, and a UV/VIS detector (Perkin Elmer Series 200) for detection.

### Data acquisition and processing

TCW (Total chrome work station version 6.3) was applied for data collection and processing. Calculations were performed with Microsoft Excel 2010.

### Validation procedure

The developed method was validated as per ICH Q2 (R1) guidelines.

### Ethics statement

Approval for HPLC estimation of DES and ALP in spiked human plasma was obtained from the Pharmacy Research Ethics committee (PREC), The Islamia University of Bahawalpur, Pakistan. “WMA Declaration of Helsinki–Ethical Principles for Medical Research Involving Human Subjects” was followed for the current study. Written informed consent was obtained from the volunteers of the study for taking their plasma. The study did not include any minor.

### Chromatographic conditions

The column utilized for the separation was a C18 reverse-phase Hypersil BDS column (250mm×4.6mm,5μm) supplied by Thermo Electron Corporation, Massachusetts, USA. The mobile phase composition utilized with this column was a mixture of acetonitrile and 0.02M potassium dihydrogen phosphate buffer (65:35 v/v) with 0.1% tri-fluoro acetic acid (TFA). The pH of the mobile phase was adjusted to 4.00 with 1M potassium hydroxide solution. The flow rate was 1.00mLmin^-1^ and the volume of injection was 20 μL. UV detection was performed at 230 nm.

### Mobile phase preparation

0.02M potassium dihydrogen phosphate was prepared by dissolving 2.72g in 100 mL de-ionized water by magnetic stirrer. Then the solution was diluted with water to 1000 mL in a 1000 mL volumetric flask. 650 mL of the buffer solution was mixed with 350 mL acetonitrile, 1000 μL TFA (0.1% of the mobile phase) was added to this mixture and stirred for 10 minutes. The original pH of this mobile phase was 2.06, which was adjusted to 4.00 by drop-wise addition of 1M potassium hydroxide solution. The mobile phase was filtered through Durapore PVDF membrane filters with a diameter of 47mm & 0.45μm pore size.

### Preparation of standard solution

To prepare a standard stock solution comprising of Desvenlafaxine (1.0 mgmL^-1^) and Alprazolam (0.5 mgmL^-1^) 100mg of DES and 50 mg of ALP drug substances were weighed and dissolved in a small volume of diluent (mobile phase) taken in 100mL volumetric flask by sonication. The volume of the solution was made up to the mark (100mL) with diluent.

Working standard solutions in the concentration range of 10–150 μgmL^-1^ for DES and 5–75 μgmL^-1^ for ALP were prepared by spiking suitable aliquots of the standard solution (7.5 mL, 5 mL, 3.5 mL, 2.5 mL, 1.875 mL, 1.25 mL, 0.5 mL) in drug-free plasma to obtain sample solutions of desired concentration of DES & ALP.

### Plasma sample preparation

Blood was obtained from healthy male human volunteers on 21-10-2019 and plasma was extracted. 1000 μL plasma was transferred to each centrifuge glass tube and various aliquots of standard drug solution of DES & ALP were added to obtain dilutions containing DES (10 μgmL^-1^, 25 μgmL^-1^, 37.5 μgmL^-1^, 50 μgmL^-1^, 75 μgmL^-1^, 100 μgmL^-1^ and 150 μgmL^-1^) and ALP (5 μgmL^-1^, 12.5 μgmL^-1^, 18.75 μgmL^-1^, 25 μgmL^-1^, 37.5 μgmL^-1^, 50 μgmL^-1^, and 75 μgmL^-1^) by addition of mobile phase to make final volume equal to 10 mL in each centrifuge tube. The tubes were vortexed for 2 minutes and then allowed to stand at room temperature for 5 minutes. Subsequently, samples were centrifuged at 4000 RPM for 10 minutes. The supernatant layer of about 3 ml was taken from each tube and filtered through nylon syringe filters (13mm & 25mm diameter and 0.45μm pore size). The samples were placed in HPLC sample vials and 20 μL of each sample was injected thrice into the HPLC column.

### Analytical method development

An isocratic reversed-phase HPLC method was adopted for a simple, specific, and scientific analysis of DES and ALP with shorter run time.

#### Selection of stationary phase (column)

During optimization of the method, various columns (Agilent C18, 200 mm×4.6 mm, 5 μm; Discovery® C8, 250 mm×4.6 mm, 5 μm Supelco; Schimadzu Shim-pack CLC-08 150 mm×4.6 mm, 5 μm; Welchrom C18, 250mm×4.6 mm, 5 μm, and Thermo Electron Corporation Hypersil BDS C18, 250mm×4.6mm, 5 μm) were tested. The well-resolved peaks of two drugs were achieved on Thermo Electron Corporation Hypersil BDS C18 250mm×4.6mm, 5 μm with acetonitrile, and 0.02M KH_2_PO_4_ buffer (65:35 v/v) with 0.1% TFA at pH 4.00.

#### Selection of mobile phase

During method development, an aqueous mobile phase with various ratios of organic modifiers (methanol & acetonitrile) was used. DES & ALP did not elute when water alone was used as the mobile phase. ALP eluted with wide peaks with methanol as the mobile phase but DES did not elute with methanol. Acetonitrile alone eluted ALP with a good response but the response of DES was quite low. Afterward, various buffers including ammonium acetate, ortho-phosphate, NaH_2_PO_4_ and KH_2_PO_4_ of various concentration values i.e. 0.01M, 0.02M, and 0.05M were tested in various ratios with methanol and acetonitrile through isocratic elution. After performing a set of screening experiments, it was deduced that 0.02M KH_2_PO_4_ buffer showed better resolution of peaks as compared to other buffers. The combination of A (a mixture of acetonitrile +methanol) and B (0.02M KH_2_PO_4_ buffer) in varying percentages was also tested. At the composition, 50% A (60% acetonitrile:40% methanol) & 50% B, both the drugs showed the same retention time. An increased percentage of methanol delayed the elution of the drugs. So methanol was excluded and a mixture of acetonitrile & buffer was optimized. Ultimately, a mixture of acetonitrile & 0.02M KH_2_PO_4_ buffer (65:35) eluted both drugs with better separation of peaks. However, peak tailing was observed which was removed by adding 0.1% TFA in the mobile phase. Hence, well-resolved and sharp peaks of DES & ALP were obtained.

The mobile phase pH influences peak shape, resolution, and symmetry. pKa value of DES is 8.87 (strongest basic) & 10.11(strongest acidic) and the pKa of ALP is 2.4. According to the rule of thumb, the mobile phase pH should be two units below or above the drug’s pKa value. If pKa of DES is considered, pH >10 could not be selected, as this pH has a detrimental influence on the column’s silica beds. Hence, pH two units below the pKa of DES could be selected. Nevertheless, concerning ALP, pH two units below its pKa (2.4) may be chosen, but it is acidic pH at which ALP completely remains in the un-dissociated state and has a strong hydrophobic binding with silica and eventually, a longer retention time. When the experiments were performed at pH 7.5, 6.5, 5.5, 4.5, and 4.0 of the mobile phase, it was observed that at low pH, both the drugs eluted with a shorter retention time as they remain in ionized form. Whereas with a rise in pH, retention of drugs increased, especially ALP eluted after 17 minutes. The effect of pH on drugs elution was also studied by variation in the strength of the buffer portion of the mobile phase from 0.01–0.05 M. 10% H_3_PO_4_ was utilized for pH reduction.

After a set of screening experiments, a combination of acetonitrile and 0.02M KH_2_PO_4_ buffer (65:35 v/v) + 0.1% TFA with pH adjusted to 4.00 with 1M KOH solution was chosen as mobile phase to give symmetric peaks with a good resolution between the drugs in shorter run time.

#### Selection of optimum flow rate

The rate of mobile phase flow through the column not only influences the retention time of the compounds but also elution. Thus, the selection of an optimized flow rate that gives well defined and resolved peaks of the analyte is of utmost necessity during the analysis. Flow rates of 0.50, 0.75, 1.0, and 1.5 mLmin*-1* were tested and 1.00 mLmin*-1* showed the sharp and symmetric peaks.

#### Selection of optimum wavelength

The selection of optimum wavelength for analysis constitutes an essential part of the analytical method development as it improves the accuracy of the quantitative analysis. Wavelength 210nm, 220nm, 230nm, 240 nm, and 250nm were tested to obtain the best response of the drugs. The optimum wavelength for detection was set because both the drugs had a higher response at 230nm.

#### Selection of diluent for standard & sample solutions

Both the drugs were dissolved in water, methanol, acetonitrile, and the mixture of organic modifier with phosphate buffer. DES and ALP were found practically insoluble in water. The drugs being sparingly soluble in methanol and acetonitrile were dissolved by sonication. But the response of the drugs in these solvents was low. The drugs solutions were formed in the optimized mobile phase with various pH i.e. 3.5, 4.0, 4.5, 5.5, 6.5, and 8.5. The best response of the drugs in terms of elution, peak shapes, and resolution was shown by the solutions of the drug in the mobile phase at pH 4.0. So the optimized mobile phase acetonitrile+0.02M KH2PO4 buffer (65:35) + 0.1% TFA, was selected as a diluent for the current study.

### Analytical method validation

The validation of the developed method was performed in terms of validation parameters i.e. system suitability, linearity, Range, accuracy, precision, detection limit, quantification limit, robustness, and stability [27].

#### System suitability

The system suitability was evaluated by injecting drug solution in six replicates containing 50 μgmL^-1^ DES and 25 μgmL^-1^ ALP and analyzing both drugs peaks for area, resolution, and tailing factor of peaks.

#### Linearity and range

A series of working standard drug solutions containing 10.0 to 150.0 μgmL*-1* for DES and 5.0 to 75.0 μgmL*-1* for ALP was formulated by diluting a suitable quantity of standard stock solution using diluent. For the calibration curve, three replicates of 20μL of each dilution were injected into HPLC promptly after preparation and the retention time was noted. The elution time and peak area of each chromatogram were evaluated. The calibration curve for each drug was plotted between the peak area of the drug on the y-axis and the corresponding drug concentration on the x-axis. After the generation of the calibration curve, a linear relationship was evaluated in terms of coefficient of determination (R*2*) within the Microsoft Excel program.

#### Accuracy

Accuracy of the method was ascertained by triplicate injections at three concentration levels (25, 50, and 75 μgmL^-1^ for DES and 12.5, 25, and 37.5 μgmL^-1^ for ALP, n = 3). The percentage recovery for each concentration was calculated by comparing the amount of drug added and the amount of drug recovered through HPLC analysis. The respective percentage recovery data for DES & ALP is summarized in Tables 2 & 3.

#### Precision

Repeatability (intra-day) and intermediate (inter-day) precision studies were performed to ensure the precision of the method. Repeatability was calculated in terms of variation among peak areas of a set of drugs solutions at levels HQC, MQC & LQC, injected in triplicate on the same day. The precision was verified from the peak area of six replicates of a reference standard solution of DES (50 μgmL^-1^) & ALP (25 μgmL^-1^). For inter-day precision, the study was carried out on three different days for the same drug solutions. The precision was interpreted in RSD value.

#### Detection limit and quantitation limit

Limit of detection (LOD) is the least level on which the drugs under study can be detected precisely and accurately. The limit of quantification (LOQ) is the least level on which the drugs under study can be quantified precisely and accurately. Various methods are available for the determination of LOD & LOQ. In the current study, LOD and LOQ were estimated from the calibration curve parameters based on the Standard Deviation of the Response and the slope, using Regression statistics in Microsoft Excel program as follows [27]:

$$LOD=3.3\times \frac{\sigma }{S}$$

$$LOQ=10\times \frac{\sigma }{S}$$

Where

σ = standard deviation of y-intercept of regression line

s = slope of calibration curve

#### Specificity

Specificity is the ability of a method to analyze analytes of interest in the presence of interfering substances likely to be present i.e. plasma proteins in this case. To assess the specificity of the developed method, three replicate injections were injected and retention times of DES & ALP were observed.

#### Robustness

Robustness of the proposed method for DES & ALP was observed by slightly changing HPLC analysis conditions including flow rate (±0.1 mLmin^-1^), pH (±1), and Acetonitrile percentage of mobile phase (±2%). Six replicates at concentration levels of DES (50 μgmL^-1^) and ALP (25 μgmL^-1^) were injected at aforesaid robust conditions.

#### Stability of analytical solution

The stability of standard as well as sample solutions was evaluated by leaving each sample and reference standard solutions of DES and ALP in screw caped HPLC vials. Short-term (bench-top) stability, Long-term stability, and Freeze-thaw stability studies were performed.

#### I. Short-term stability (bench-top stability)

Three aliquots of each LQC (10 μgmL^-1^ DES & 5 μgmL^-1^ ALP) and HQC (100 μgmL^-1^ DES & 50 μgmL^-1^ ALP) samples were kept at the ambient temperature being protected from light. To evaluate the stability of the solutions, each drug in sample solutions was quantitatively determined at various time intervals and analyzed at HPLC. The sample solution was injected after every 2 hours interval of up to 24 hours.

#### II. Long-term stability

Three aliquots of each LQC and HQC samples containing DES & ALP were frozen at 8°C and samples were analyzed at intervals of 15 days up to 60 days.

#### III. Freeze-thaw stability

The freeze-thaw stability of studied sample solutions was evaluated at LQC and HQC levels over 3 cycles within three days. The spiked plasma samples were stored in screw-capped HPLC vials at 4˚C for 24 h. For each cycle, the frozen spiked plasma samples were thawed at room temperature for 2 h. The samples were thawed completely and refrozen afterward for 24 h under the same storage conditions. After completion of each freeze-thaw cycle, the samples were analyzed on HPLC and the results were compared with those of the zero cycle.

## Results

### Method development and optimization

#### Optimized chromatographic conditions

Several HPLC chromatographic conditions were investigated for optimization of the conditions to analyze DES & ALP in a single run. Optimized chromatographic conditions for this developed HPLC method along with the retention time for DES & ALP are shown in Table 1.

**Table 1:** Proposed chromatographic conditions for DES & ALP.

Parameters	Condition
Mobile phase	Acetonitrile: 0.02M KH_2_PO_4_ buffer (65:35 v/v)+ 0.1% TFA, pH adjusted to 4.00
Stationary phase (column)	Hypersil BDS C_18_ (250mm × 4.6 mm,5 μm)
Column temperature	Ambient
Flow rate	1.00 mLmin^-1^
Run time	12 min
λmax	230 nm
Volume of sample injected	20 μL
Retention time	4.011 min (DES) and 5.182 (ALP) min

**Calibration curve.** A series of working standard drug solutions containing 10.0 to 150.0 μgmL^-1^ for DES and 5.0 to 75.0 μgmL-1 for ALP was formulated by diluting a suitable quantity of standard stock solution using diluent. For the calibration curve, three replicates of 20μL of each dilution were injected into HPLC promptly after preparation and the retention time was noted. The elution time and peak area of each chromatogram were evaluated. The calibration curve for each drug was plotted between the peak area of the drug on the y-axis and the corresponding drug concentration on the x-axis. The evaluation of the calibration curve was carried out in terms of its coefficient of determination (R^2^).

### Analytical method validation

Various parameters including selectivity, system suitability, linearity and range, precision, accuracy, LOD, LOQ, specificity, robustness, and stability of analytes were evaluated for validating the developed analytical method as per ICH Q2(R1) guidelines [27].

#### System suitability

**Fig 3:**
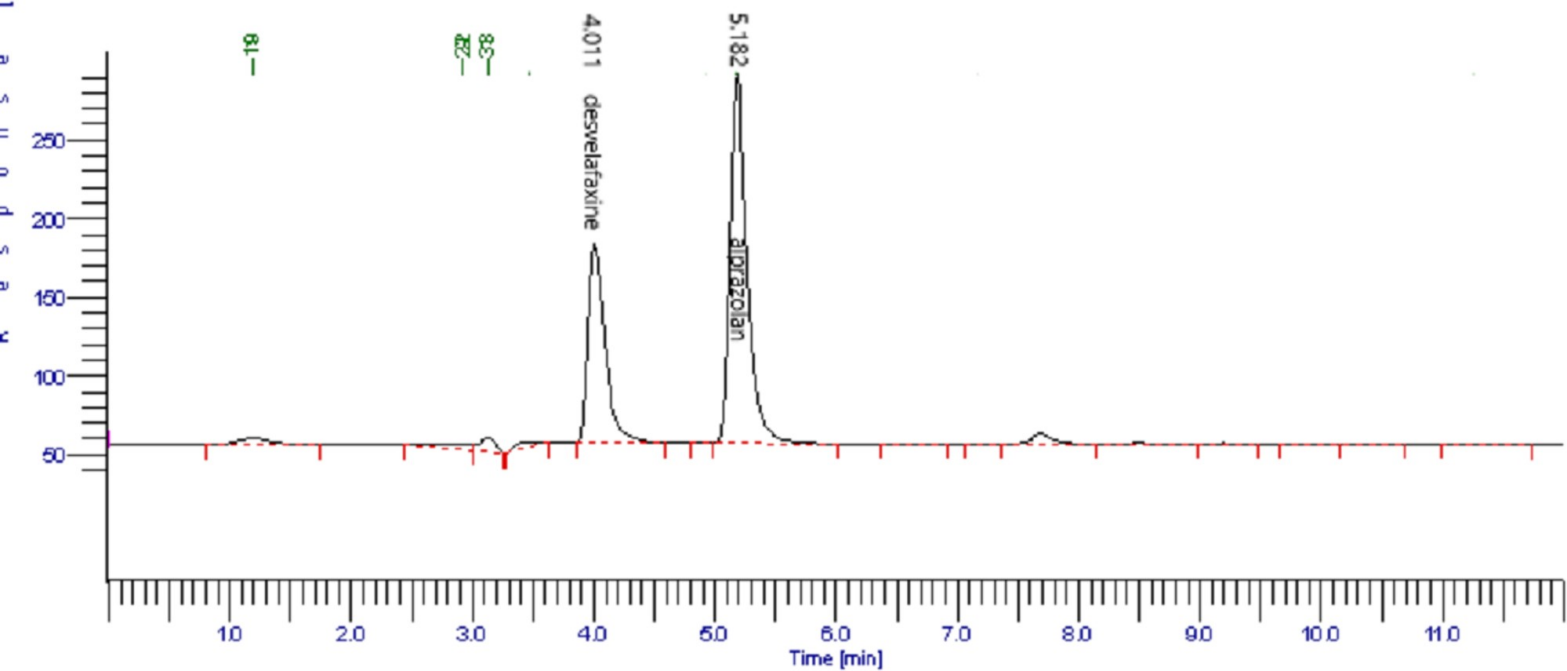
Representative chromatogram of human plasma spiked with DES (100 μgmL^-1^) and ALP (50 μgmL^-1^) with elution time 4.011 min & 5.182 min respectively.

The acceptance criteria of a developed analytical method for system suitability parameters were %RSD for peak areas <2%, tailing factor of peak <1.5, and resolution >2.0 among adjacent peaks of drugs. The results obtained showed that all system suitability parameters for the method conformed to the acceptance criteria. The resolution among DES and ALP and other eluting peaks was >4 which assured reliability of this method for the quantification of DES & ALP simultaneously. Fig 3 shows a typical chromatogram of human plasma spiked with DES & ALP.

#### Selectivity

Selectivity is the capability of the method to differentiate, identify, and analyze analytes in the presence of other components in the sample. Spiked plasma samples of LQC, MQC, and HQC were chromatographed to check the interference of endogenous compounds during the analysis of DES and ALP. Peaks of DES and ALP did show any interference of endogenous components and a very good resolution was observed between peaks of DES and ALP. The resolution between DES and ALP is 4.3 and it confirms the selectivity of the method.

#### Linearity and range

The linearity of a given method is its ability to give analysis results in direct proportion to the amount of analyte in the sample (within a given range). The individual calibration curve of DES & ALP was plotted over the concentration ranging from 10–150.0 μgmL^-1^ for DES and 5–75.0 μgmL^-1^ for ALP respectively. It was observed that DES had linearity in the range of 10–150.0 μgmL^-1^ with linear regression equation (y = 23.517x+38.759, R^2^ = 0.9983) and ALP had linearity in the range 5–75.0 μgmL^-1^ with linear regression equation (y = 143.43x+182.09, R^2^ = 0.9989). Correlation coefficient was > 0.9980 as shown in the Table 2.

**Table 2:** Calibration data for estimation of DES & ALP in human spiked plasma.

Parameter	Optimum conc. range (μgmL^-1^)	Correlation coefficient	Limit of detection (μgmL^-1^)	Limit of quantification (μgmL^-1^)
Desvenlafaxine	10–150.0	0.9983	4.740	14.365
Alprazolam	5–75.0	0.9989	1.891	5.730

#### Accuracy & precision

The accuracy of the developed method was evaluated from the recovery test. After repeating the assay for three consecutive days, resultant %RSD and % recovery values observed for DES and ALP are shown in Tables 3 & 4 Percent recovery values obtained during this method fall in the range of 99 to 101.88%.

**Table 3:** Accuracy & precision studies of desvenlafaxine.

Drug	Level	Drug Conc. (μgmL^-1^)	Intra-day precision			Inter-day precision		
			*Quantified conc. ± SD (μgmL^-1^)	RSD (%)	*Recovery (%)	*Quantified conc. ± SD (μgmL^-1^)	RSD (%)	*Recovery (%)
DES	LQC	25	24.98±0.035	0.361	99.92	25.47±0.113	0.327	101.88
	MQC	50	49.84±0.206	0.413	99.680	49.80±0.228	0.458	99.600
	HQC	75	74.71±0.196	0.275	99.613	74.80±0.664	0.692	99.733

* Mean of three replicates.

**Table 4:** Accuracy and precision studies of alprazolam.

Drug	Level	Drug Conc. (μgmL^-1^)	Intra-day precision			Inter-day precision		
			*Quantified conc. ± SD (μgmL^-1^)	RSD (%)	*Recovery (%)	*Quantified conc. ± SD (μgmL^-1^)	RSD (%)	*Recovery (%)
ALP	LQC	12.5	12.69±0.042	0.341	101.52	12.39±0.162	0.536	99.120
	MQC	25	24.99±0.043	0.175	99.997	25.015±0.111	0.447	100.066
	HQC	37.5	37.27±0.231	0.483	99.386	37.79±0.395	0.762	100.77

* Mean of three replicates.

Precision results were interpreted as RSD values of drug peaks. RSD values obtained for peak areas of DES on a single day for intra-day precision were from 0.275–0.413% and for ALP 0.175–0.483%. For inter-day precision, triplicate injections of drug solution on three successive days (days 1–3) showed %RSD value 0.327–0.692 for DES and 0.447–0.762 for ALP. Precision data for DES & ALP is presented in Tables 3 & 4

#### LOD & LLOQ

The minimum amount of DES & ALP detected by the currently developed method in human spiked plasma was 4.740 μgmL*-1* for DES & 1.891 μgmL*-1* for ALP whereas minimum amounts of these drugs quantified by the method, that is, LOQ, were 14.365 μgmL*-1* & 5.730 μgmL*-1* for DES &ALP respectively as shown in the Table 2.

#### Robustness

To interpret robustness, the method was performed with deliberate variations in analytical parameters. The results for the assay of DES & ALP obtained by this variation in analytical conditions were not affected and found to conform to results observed with optimized conditions. No significant variation in the chromatograms of both drugs was observed by variation of optimized chromatographic conditions. % RSD value of assay results of sample solutions under original optimized conditions & robustness conditions was < 2.0%.

#### Stability of sample solutions

Tables 5 & 6 exhibit a summary of stability of DES & ALP in human spiked plasma at conditions of Short-term stability, Long-term stability, and Freeze-thaw cycles. Samples were stored at room temperature and were injected after every 2 hours up to 24 hours to observe the stability of the solution. During freeze-thaw cycles, freezing and thawing of samples at regular cycles were followed by injecting into HPLC. For long term stability study samples were stored at -20°C and were evaluated up to 60 days.

**Table 5:** Results of Stability study of DES in human spiked plasma.

QC Level	Short-term (Bench-top) stability at Room temperature			Long term stability		Freeze-thaw stability	
	Nominal (μgmL^-1^)	*Mean (Conc. Found)	RSD %	*Mean	RSD %	*Mean	RSD %
LQC	10	9.987	0.0917	9.695	0.0469	9.798	0.0441
MQC	50	49.991	0.0359	49.985	0.0172	49.728	0.0913
HQC	100	99.897	0.0380	99.935	0.0469	99.875	0.0106

* Mean of three replicates.

**Table 6:** Results of Stability study of ALP in human spiked plasma.

QC Level	Short-term (Bench-top) stability at Room temperature			Long term stability		Freeze-thaw stability	
	Nominal (μgmL^-1^)	*Mean (Conc. Found)	RSD %	*Mean (Conc. Found)	RSD %	*Mean (Conc. Found)	RSD %
LQC	5	4.989	0.134	4.798	1.05	4.896	0.762
MQC	25	24.997	0.258	24.674	1.751	24.978	0.653
HQC	50	49.992	0.064	49.442	0.4634	49.985	0.359

* Mean of three replicates.

## Discussion

The current study reports a simple, rapid, robust, precise, accurate, and validated RP-HPLC method for simultaneous determination of DES & ALP in human spiked plasma which was validated in consonance with ICH guidelines [27]. All parameters of system suitability for peaks of both the drugs conformed to the acceptance criteria that ensured aptness of the current method for simultaneous quantification of DES & ALP in human spiked plasma.

The linearity and range study for DES & ALP revealed that the response of both drugs was linear from 10–150.0 μgmL^-1^ for DES and 5.0–75.0 μgmL^-1^ for ALP. The coefficient of determination was greater than 0.9980 for both drugs. In this method, the LOD of DES & ALP was 4.740 μgmL^-1^ & 1.891 μgmL^-1^, respectively. LOQ determined as per ICH guidelines was found to be 14.365 μgmL^-1^ for DES and 5.730 μgmL^-1^ for ALP.

The % RSD for accuracy and precision studies for DES and ALP was found to be <3.0%. The method is accurate, as evident from excellent recovery during the recovery study. Accuracy and precision study implies that the developed method is highly precise as well as accurate for the simultaneous determination of DES and ALP.

The chromatographic parameters of the method did not show significant variation when performing the experiments with varied experimental conditions including flow rate, wavelength, and pH of the mobile phase. %RSD value for assay of the same samples of DES & ALP for robustness experiments did not exceed 2%, demonstrating that the developed method is robust.

The results of sample solution Short-term stability, Long-term stability, and freeze-thaw stability as shown in Table 4 & 5 imply that retention times and peak areas of DES & ALP had RSD <1 and that no remarkable degradation was observed in sample solutions within the stated period, as no change in drug elution behavior and peak shape was observed. It demonstrates that the analyte solutions are stable in solution state at these conditions for two months and can, therefore, be used within this period without the results being affected.

The reported method is very simple and requires a run time of about 12 minutes, that is, 2.5 times of retention time of analytes detected by the method. As it has a reduced analysis time, so it can minimize the incurred cost of DES and ALP analysis as compared to the methods available for individual estimation of both drugs. The reported method is specific, efficient as well as cost-effective for simultaneous determination of DES and ALP in spiked human plasma without any interference from the endogenous components present in plasma (i.e. plasma proteins).

## Conclusion

Conclusively, the reported RP–HPLC method is simple, precise, accurate, robust, and economical with a UV detector involving no derivatization or other expensive technique. The preparation of plasma samples requires a very simple single-step protein precipitation that makes the method easier and faster. DES and ALP were simultaneously quantified with good resolution and retention time of 4.011 minutes of DES and 5.182 minutes of ALP. The method is linear with R^2^ >0.9980 and has a good analytical range for both drugs (10–150 μgmL^-1^ for DES and 5–75 μgmL^-1^ for ALP). This method utilizes a simple mobile phase that is easy to prepare. All tests carried out for the validation of the developed method are in full conformity with the acceptance criteria.

This method owns a great promise as a rapid analytical tool for the simultaneous estimation of DES & ALP with good separation and resolution in a single chromatographic run (12 minutes) and can be used for estimation of DES and ALP in plasma and can evaluate pharmacokinetic parameters of both drugs simultaneously.
